# Neonatal high pressure hydrocephalus is associated with elevation of pro-inflammatory cytokines IL-18 and IFNγ in cerebrospinal fluid

**DOI:** 10.1186/1743-8454-5-21

**Published:** 2008-12-31

**Authors:** Deborah A Sival, Ursula Felderhoff-Müser, Thomas Schmitz, Eelco W Hoving, Carlo Schaller, Axel Heep

**Affiliations:** 1 Department of Pediatrics, University Medical Center Groningen, University of Groningen, Hanzeplein 1, 9700RB Groningen, The Netherlands; 2 Department of Kinderheilkunde Zentrum für Kinder- und Jugendmedizin, Universitätsklinikum Essen, Hufelandstrasse 55, D-45122 Essen, Germany; 3 Department of Neonatology, Campus Virchow Klinikum, Charité – Universitätsmedizin Berlin, 13353 Berlin, Germany; 4 Department of Neurosurgery, University Medical Center Groningen, University of Groningen, Hanzeplein 1, 9700RB Groningen, The Netherlands; 5 Department of Neurosurgery, University of Bonn Medical Center, Sigmund-Freud-Str 25, 53127 Bonn, Germany; 6 Department of Neonatology, University of Bonn, Adenauerallee 119, 53113, Bonn, Germany

## Abstract

**Background:**

In human neonatal high pressure hydrocephalus (HPHC), diffuse white matter injury and gliosis predispose to poor neuro-developmental outcome. The underlying mechanism for diffuse white matter damage in neonatal HPHC is still unclear. Analogous to inflammatory white matter damage after neonatal hypoxemia/ischemia, we hypothesized that pro-inflammatory cytokines could be involved in neonatal HPHC. If so, early anti-inflammatory therapy could ameliorate white matter damage in HPHC, before irreversible apoptosis has occurred. In HPHC and control neonates, we therefore aimed to compare cerebrospinal fluid (CSF) concentrations of IL18, IFNγ and sFasL (interleukin 18, interferon gamma and apoptosis marker soluble-Fas ligand, respectively).

**Methods:**

In neonatal HPHC (n = 30) and controls (n = 15), we compared CSF concentrations of IL18, IFNγ and sFasL using sandwich ELISA. HPHC was grouped according to etiology: spina bifida aperta (n = 20), aqueduct stenosis (n = 4), and fetal intra-cerebral haemorrhage (n = 6). Neonatal control CSF was derived from otherwise healthy neonates (n = 15), who underwent lumbar puncture for exclusion of meningitis.

**Results:**

In all three HPHC groups, CSF IL18 concentrations were significantly higher than control values, and the fetal intracranial haemorrhage group was significantly higher than SBA group. Similarly, in all HPHC groups CSF-IFNγ concentrations significantly exceeded the control group. In both HPHC and control neonates, CSF FasL concentrations remained within the range of reference values.

**Conclusion:**

Independent of the pathogenesis, neonatal HPHC is associated with the activation of the pro-inflammatory cytokines (IL-18 and IFNγ) in the CSF, whereas CSF apoptosis biomarkers (sFasL) were unchanged. This suggests that anti-inflammatory treatment (in addition to shunting) could be helpful to preserve cerebral white matter.

## Background

Since the introduction of innovative drainage valves and third ventricular endoscopy, neurosurgical treatment strategies for neonatal HPHC have improved. Nevertheless, HPHC is still associated with irreversible white matter damage and adverse neurological outcome [1-4]. After hypoxemia/ischemia, white matter damage consists of a diffuse, inflammatory pattern involving pro-inflammatory cytokines, oligodendrocytic injury, gliosis and myelin loss [5-7]. Pro-inflammatory cytokines are biologically active proteins produced by T cells, astrocytes and microglial cells. After cytokine release, immune cells invade the brain and subsequently activate astrocytes and microglial cells, which results in apoptosis and gliosis [5,8].

Especially, the immature central nervous system is vulnerable for inflammatory damage. This is attributed to the specific sensitivity of immature oligodendrocytes for microglial cells, glutamate and free radicals [9,10]. Although shunting will improve cerebral perfusion and prevent gliosis [11], shunting does not address inflammatory consequences. Thus, long-acting cytokines (released before shunting), could theoretically continue to damage oligodendrocytes after shunting [6,12]. In the neonatal CNS, inflammatory mechanisms may contribute to diffuse white matter damage not only in the periventricular regions, but also at a distance from the ventricles [9]. Hypoxemia/ischemia is associated with cytokine IL18 release and cystic white matter damage [6]. Cytokine IL18 can induce other pro-inflammatory cytokines, such as IFNγ, IL-1β and TNFα [13]. In contrast to elevated CSF IL18 concentrations which last for months, IL-1β and TNFα concentrations are only elevated for hours [14,15]. This may explain our previously reported negative association between CSF IL-1β concentration and cystic white matter damage [6]. Cytokine IFNγ is also involved in the regulation of the inflammatory response by activation of cytotoxic T-cells and macrophages [16]. Upon activation, this may result in apoptosis, myelin loss and gliosis [5,12,17-20]. In neonates with post-hemorrhagic hydrocephalus and cystic white matter damage, we have subsequently shown that enhanced growth factor concentrations (i.e. vascular endothelial growth factor (VEGF) and transforming growth factor β1 (TGF-β1)) will finally reflect tissue repair [21,22].

In this perspective, we hypothesized that hypoxemia/ischemia-related up-regulation of longer acting cytokines (IL18 and IFNγ) could be involved in neonatal white matter damage by HPHC. If cytokines are involved in ongoing white matter damage, early anti-inflammatory therapy could be beneficial, before irreversible apoptosis has occurred. The apoptosis biomarkers Fas and Fas-ligand (FasL) are members of the tumour-necrosis factor super family. The death Fas (CD95/Apo-1) is located on the cell surface. It plays a pivotal role in transduction of the apoptotic cell death program. Fas and its FasL exist in membrane bound form and soluble forms and can be detected in neonatal CSF [23]. Soluble FasL (sFasL) is expressed on activated T cells and released by metalloproteinase. SFasL can regulate extracellular apoptosis by pro- and anti-apoptotic properties. Expression of sFasL indicates ongoing apoptosis.

In neonatal HPHC characterized by progressive ventriculomegaly and increased head circumference > P_75_, and control patients, this study aimed to determine and compare CSF IL18, IFNγ and sFasL concentrations. We hypothesized that CSF IL-18 and IFNγ concentrations are increased in HPHC, irrespective of underlying etiology. To investigate this, HPHC patients were grouped according to three different aetiologies: spina bifida aperta (SBA), aqueduct stenosis, and hydrocephalus after fetal intra-cranial haemorrhage.

## Methods

### Patients selection and CSF sampling

The study was approved by the medical ethical committees of the University of Bonn, the Charité Universitäts Medizin Berlin and the University Medical Center Groningen. After informed consent by the parents, 30 HPHC and 15 control neonates were included. In neonatal HPHC, CSF was obtained during initial neonatal shunt surgery. Indications for shunting consisted of clinical signs for high intracranial pressure, bulging fontanel, widening of the sagittal suture, progressive ventriculomegaly and increased head circumference (> P_75_). Since anaesthesia, artificial respiration and internal pressure compensation may quantitatively influence the assessment of intracranial pressure, CSF pressure was not measured routinely during shunt placement. Neonatal HPHC was grouped according to aetiology: SBA (n = 20; characterized by presence of meningomyelocele), aqueduct stenosis (n = 4); HC after fetal intra-cranial haemorrhage (n = 6). Selection of HC after fetal intra-cranial haemorrhage (i.e. haemorrhage 4–6 weeks before delivery) allowed avoidance of the potentially confounding influence by disintegration of platelets. The diagnosis of fetal post-hemorrhagic hydrocephalus was confirmed by prenatal ultrasound (ATL 500, 3.5 MHz transducer), postnatal ultrasound (Vingmed Vivid5, multi-frequency transducer (5–7.5–10 MHz crystals) and magnetic resonance imaging (Philips Healthcare, Best, Netherlands, 1.5 Tesla). Low risk neonates, undergoing lumbar puncture for exclusion of meningitis, served as controls (n = 15). Gestational ages in the three hydrocephalic groups and control group were similar, i.e. between 27–54 and 24–54 weeks, respectively. CSF samples obtained during shunt revisions, neonatal asphyxia and CNS infections were excluded. Cerebral infection was excluded by negative CSF cultures, cellular count, total protein concentration and by assessment of CSF-IL6 concentrations (in CNS infection, CSF IL-6 concentrations are increased). CSF-IL6 concentrations were measured by commercially available solid-phase enzyme-labelled chemiluminescent sequential immunometric assay on an Immulite analyzer (DPC Biermann, Bad Naunheim, Germany). All CSF IL-6 concentrations were within the normal range 5 pg/ml – 200 pg/ml (i.e. far below CSF IL-6 levels in newborns with bacterial ventriculitis [24]). Total CSF protein content varied between 0.1 – 2.5 g/l.

### CSF analysis

All CSF samples were immediately centrifuged and stored at -40°C for further analysis. CSF concentrations of IL-18 and IFNγ were determined by sandwich ELISA (R&D systems, Wiesbaden, Germany) according to the manufacturer's instructions. The sensitivity of the assay was 12.5 pg/ml for IL18, 8.0 pg/ml for IFNγ and 0.5 ng/ml for sFasL using Mab for coating and binding (clones 4H9 and 4A5) [6,23]. The intra-assay coefficient of variation was 5.0% for IL18 and 4.7% for IFNγ. All ELISA 96-well micro titer plates were analyzed using a microplate photometer (Dynotech MR5000, Denkendorf, Germany). Neonatal control CSF sFasL data were derived from our previous study by application of the same analytical technique, performed by the same laboratory [23].

### Data analysis

For statistical analysis, Mann-Whitney U test was used with two-sided p values to compare continuous nonparametric group of values, as the distribution of values was non-Gaussian.

## Results

**Figure 1:**
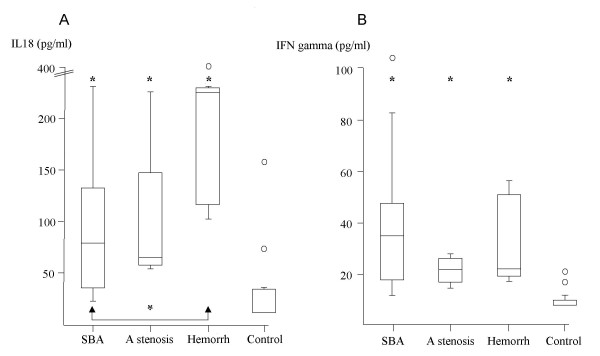
**(A) Graphs of CSF interleukin-18 (IL-18) concentration and (B) CSF interferon gamma (IFN gamma) concentration in CSF from neonatal HPHC. The vertical axes indicate concentration (pg/ml)**. The horizontal axes indicates three different age-matched aetiologies for neonatal HPHC: spina bifida aperta (SBA), aqueduct stenosis (A stenosis), and fetal intracranial hemorrhage (Hemorrh) and neonatal controls (Control). Data are median and range plus 25^th ^and 75^th ^percentiles Encircled symbols in the figures indicate single parameters that appeared out of range. A: In all three neonatal HPHC groups, IL-18 concentrations were significantly higher than in controls (* *p *< 0.01). Furthermore, the fetal intracranial hemorrhage hydrocephalus group was significantly higher than the SBA hydrocephalus group (indicated by arrows at the bottom * *p *< 0.01). B: In all three neonatal HPHC groups, CSF IFNγ concentrations were significantly higher than in controls (* *p *<*0.01*).

Irrespective of the underlying cause, IL-18 concentrations were significantly higher in HPHC neonates than in controls, median and range: SBA: 80 (23–232) pg/ml; aqueduct stenosis: 66 (55–226) pg/ml; fetal intracranial haemorrhage: 223 (103–406) pg/ml; controls: 12.5 (12.5–158) pg/ml. Each group was significantly higher than control, *p *< 0.01, and the fetal intracranial haemorrhage group was significantly higher than SBA, *p *< 0.01; figure 1A). Similarly, CSF IFNγ concentrations were also significantly higher in the three HPHC groups than in controls, median and range: SBA: 35 (12–139) pg/ml; aqueduct stenosis: 22 (15–28) pg/mL; fetal intracranial haemorrhage: 22 (17–56) pg/mL; controls: 8 (8–22) pg/ml. Each group was significantly higher than controls, *p *< 0.01; but not significantly different between the groups (figure 1B). In all three neonatal HPHC groups, CSF sFasL concentrations remained within control limits, < 0.5 ng/ml [23].

## Discussion

Under diverse cerebral pathological circumstances, both astroglial and microglial alterations may be involved in white matter damage and adverse neurological outcome. It is indicated that hydrocephalus-associated brain tissue compression can instigate proliferation of astrocytes and microglial cells resulting in gliosis [11]. This study has shown that irrespective of the underlying aetiology, early indications for HPHC (derived from concurring ventriculomegaly and macrocephaly) are accompanied by pro-inflammatory cytokine activation (IL-18 and IFNγ) with highest IL18 concentrations in post-hemorrhagic HPHC. Despite cytokine release into the CSF, the CSF concentrations of the apoptosis biomarker sFasL remained within control limits. These results are contrasted by our previous findings of high CSF sFasL concentrations in neonatal cystic white matter damage [23]. In the present study, normal CSF sFasL concentrations are explained by early assessment of CSF samples during the first shunt implantation and before cystic white matter alterations have occurred. All together in early neonatal HPHC, present data indicate that inflammation precedes irreversible apoptosis, which may provide a theoretical basis for early anti-inflammatory therapy (at about the time of first shunt implantation). In children with leucomalacia and post-haemorrhagic hydrocephalus, similar cytokine activation is associated with a diffuse component of white matter damage, prolonged myelination delay (for months) and even permanent myelin deficiency [5,6]. From a neuro-pathological point of view, concurrent white matter lesions of varying appearance and age (acute, organizing and chronic) suggest various, ongoing insults in the same patient [5]. However, before these data can be extrapolated to all groups of neonatal HPHC, histological examination (by immunostaining) will be required. Analogous to pediatric HPHC, adult patients with normal pressure hydrocephalus and/or vascular dementia may also have elevated pro-inflammatory cytokine concentrations (TNFα) in association with white matter damage [25,26]. However, because of patient heterogeneity, age-specific cytokine sensitivity and variability in disease progression, it is not possible to speculate further about similarities in inflammatory involvement between ages.

In neonatal H-Tx rat (i.e. an animal model for congenital hydrocephalus by aqueduct stenosis), it was shown that shunting could ameliorate gliosis [11]. Since gliosis may be associated with both reactive astrocytosis and microgliosis, one would expect that anti-inflammatory therapy could have a beneficial effect in addition to shunting. Accordingly, it was shown that minocycline, a semi-synthetic second generation tetracycline with anti-inflammatory, anti-apoptotic and anti-glutaminergic properties [27], reduces gliotic scarring in H-Tx rat [28]. Although minocycline is contra-indicated in young children, present human neonatal HPHC data suggest that other anti-inflammatory compounds could theoretically ameliorate diffuse cytokine-coupled, white matter damage [11]. In multiple sclerosis (characterized by up-regulation of pro-inflammatory cytokines), different anti-inflammatory agents (such as interferon beta (IFNβ) and glatiramer acetate) are known to ameliorate white matter damage [29]. Although there may be a rational basis for early neonatal (or perhaps even fetal) application of such anti-inflammatory compounds, potentially harmful adverse reactions should first be considered.

## Conclusion

Neonatal HPHC irrespective of cause, is accompanied by pro-inflammatory cytokine activation (IL-18 and IFNγ) in the CSF. These data suggest that anti-inflammatory treatment (in addition to shunting) could be helpful to preserve cerebral white matter in these patients.

## Abbreviations

CSF: cerebrospinal fluid; GA: gestational age; HPHC: neonatal high pressure hydrocephalus; IFNβ: interferon beta; IFNγ: interferon gamma; IL18: interleukin 18; sFas: soluble Fas ligand; SBA: spina bifida aperta.

## Competing interests

The authors declare that they have no competing interests.

## Authors' contributions

DS contributed to the study design, collection of CSF and writing of the manuscript. UF contributed to the study design, assessment of CSF samples and correction of the manuscript. TS contributed to the assessment of CSF samples and correction of the manuscript. EH contributed to collection of CSF and correction of the manuscript. CS contributed to collection of CSF and correction of the manuscript. AH contributed to the study design, collection of CSF, statistical analysis, and helped drafting and correcting the manuscript. All authors have read and approved the final version of the manuscript.
